# The Paradoxical Immunomodulatory Effects of Chitosan in Biomedicine

**DOI:** 10.3390/polym17010019

**Published:** 2024-12-25

**Authors:** Sophie L. Reay, Ana Marina Ferreira, Catharien M. U. Hilkens, Katarina Novakovic

**Affiliations:** 1 School of Engineering, Newcastle University, Newcastle Upon Tyne NE1 7RU, UK; ana.ferreira-duarte@newcastle.ac.uk (A.M.F.); katarina.novakovic@newcastle.ac.uk (K.N.); 2 Translational & Clinical Research Institute, Newcastle University, Newcastle upon Tyne NE1 7RU, UK; catharien.hilkens@newcastle.ac.uk

**Keywords:** chitosan, endotoxin, immunomodulation, pro-inflammatory, anti-inflammatory

## Abstract

Chitosan is widely explored in the field of biomedicine due to its abundance and reported properties, including biocompatibility, biodegradability, non-toxicity, mucoadhesion, and anti-microbial activity. Although our understanding of the immune response to chitosan has evolved, confusion remains regarding whether chitosan is a pro- or anti-inflammatory biomaterial. Tackling this knowledge gap is essential for the translation of chitosan-based biomaterials to clinical use. Herein, we provide an overview of the immune responses to chitosan, exploring the roles of endotoxin contamination and physiochemical properties in immunomodulation. Ultimately, this literature review concludes that various physiochemical properties, including molecular weight, degree of deacetylation and polydispersity, endotoxin contamination, and cellular environment, interplay in the complex process of chitosan immunomodulation, which can lead to both pro- and anti-inflammatory effects.

## 1. Introduction

**Figure 1:**
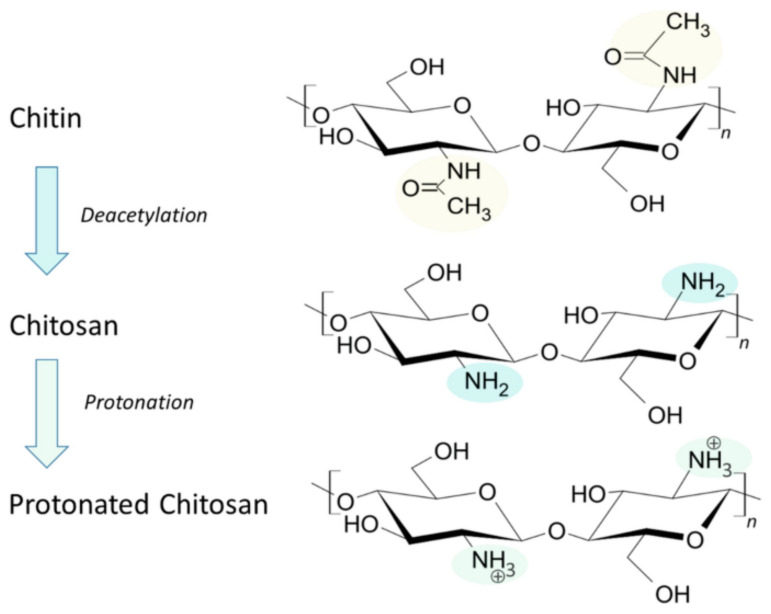
Structure of chitin and chitosan. Deacetylation of chitin produces chitosan, converting the acetyl groups (highlighted in yellow) to amino groups (highlighted in blue), which can subsequently become protonated (highlighted in green) in acidic conditions. Adapted from [16].

Chitosan is a natural, cationic, linear copolymer composed of randomly distributed N-acetyl-D-glucosamine and D-glucosamine units [1] (Figure 1). Chitosan is derived from chitin, the second most abundant natural polymer after cellulose [2]. Chitin can be extracted from a plethora of natural resources, such as marine, microbial, and terrestrial sources [3]. As a major by-product of the fishing industry, crustacean shells are a particularly abundant source of chitin, with the annual production of shell waste by-products exceeding 10 million tons [4]. As a renewable material, chitin is gaining much attention due to the global sustainability drive [5,6,7,8]. Despite its abundance, chitin has limited applications due to its crystalline structure and inability to dissolve in most solvents. Therefore, chitin is often converted to its more soluble derivative, chitosan, via partial or full deacetylation, whereby hydrophobic acetyl groups are converted to hydrophilic amino groups. Although both acids and alkalis can deacetylate chitin, alkalis are more often used because the glycosidic bond is susceptible to degradation by acid [9]. An alternative method is enzymatic deacetylation using chitin deacetylase from biological sources, including fungi and insects. Chitin with a degree of deacetylation (DD) above 50% is classified as chitosan [10], with most commercial chitosan having DD values between 70% and 90% [11]. With a pKa of approximately 6.5, chitosan is soluble in acidic solutions owing to the protonation of amino groups present in the polymeric backbone. The solubility of chitosan is therefore a function of DD, with highly deacetylated chitosan having enhanced solubility [12]. The extent of deacetylation also affects additional physiochemical properties, including biodegradation [13] and electrostatic properties [14]. However, the intermolecular and intramolecular hydrogen bonds of chitosan lead to crystalline structures, making it insoluble in water and limiting its biomedical applications. Chitosan derivatives can be obtained through the chemical modification of chitosan’s active functional groups, including acylation, carboxylation, and alkylation. The addition of hydrophilic groups improves the water solubility of chitosan, thus expanding its potential applications [15].

Chitosan can undergo enzymatic degradation in the presence of several enzymes, including chitinases and proteases [17]. Biodegradability is a paramount consideration for biomedical biomaterials, as it avoids the need for invasive surgical removal, thus making these materials more acceptable for patients, enhancing the likelihood of obtaining regulatory approval, as well as reducing treatment costs for the health service. In the human body, chitosan is mainly degraded by lysozyme, which is detected in bodily secretions, including tears, saliva, and serum [18]. Lysozyme primarily functions as an antimicrobial agent by hydrolysing the β-(1,4) linkages between N-acetylmuramic acid and N-acetylglucosamine in the peptidoglycan wall of Gram-positive bacteria [19,20]. As chitosan has a similar structure, consisting of N-acetyl-D-glucosamine and D-glucosamine units, it is possible that lysozyme degrades chitosan using a similar mechanism. Lysozyme only interacts with the acetylated units of chitosan [21,22]; thus, lysozyme digestibility decreases with the increase in DD [23,24,25,26].

Due to distinctive qualities, including abundance, biocompatibility, biodegradability, non-toxicity, mucoadhesion and antimicrobial activity [27], chitosan is widely applied in the realms of biomedicine. The functional groups present in the chitosan backbone, namely hydroxyl and amino groups, allow the synthesis of hydrogels, which are defined as three-dimensional (3D) physically or chemically crosslinked hydrophilic polymers that largely consist of water. The versatile network architecture of chitosan-based hydrogels and the ability to incorporate various bioactive molecules enable a broad range of biomedical applications, including drug delivery, wound healing, and tissue engineering. Due to its cationic nature, chitosan is uniquely mucoadhesive, making it ideal for oral, ocular, nasal, and vaginal drug delivery [28]. Furthermore, by virtue of its pH-responsiveness, chitosan-based hydrogels are extremely promising vehicles for site-specific gastrointestinal drug delivery [29]. A novel application of chitosan is in gene delivery, whereby genetic material, such as DNA or RNA, is electrostatically linked to chitosan and introduced into cells to modify their function [9]. Chitosan is also extensively used in vaccine delivery. Not only does chitosan readily bind to negatively charged antigens or genetic material, protecting them from degradation, but it also has intrinsic adjuvanticity [26,30]. Chitosan hydrogels are ideal materials for wound dressings due to their antimicrobial, biologically adhesive, biologically active, and haemostatic effects [31]. Furthermore, chitosan is well renowned for its antioxidant activity, as its functional groups can scavenge unstable free radicals [32]. Chitosan has fundamental properties that meet the criteria for tissue engineering, including being biocompatible with tissues; possessing a cationic surface charge that promotes cell attachment, differentiation, and proliferation; being degradable by several enzymes in vivo; and possessing tuneable properties, including mechanical strength, porosity and morphology. As a result, chitosan has been studied for a multitude of tissue engineering applications, including bone, cartilage, intervertebral disk, blood vessel, and corneal and skin tissue engineering [33,34]. The paradoxical antimicrobial, antioxidant, and adjuvant bioactivities of chitosan remain highly confusing and require investigation.

This review critically examines the host immune response to biomaterials, focusing on divergent reports of chitosan-induced pro- and anti-inflammatory responses and pathways. Endotoxin contamination of chitosan is considered, as well as the role of various physiochemical properties and cellular environment on immunomodulation.

## 2. Immune Response to Biomaterials

### 2.1. The Role of Dendritic Cells in the Immune Response

**Figure 2:**
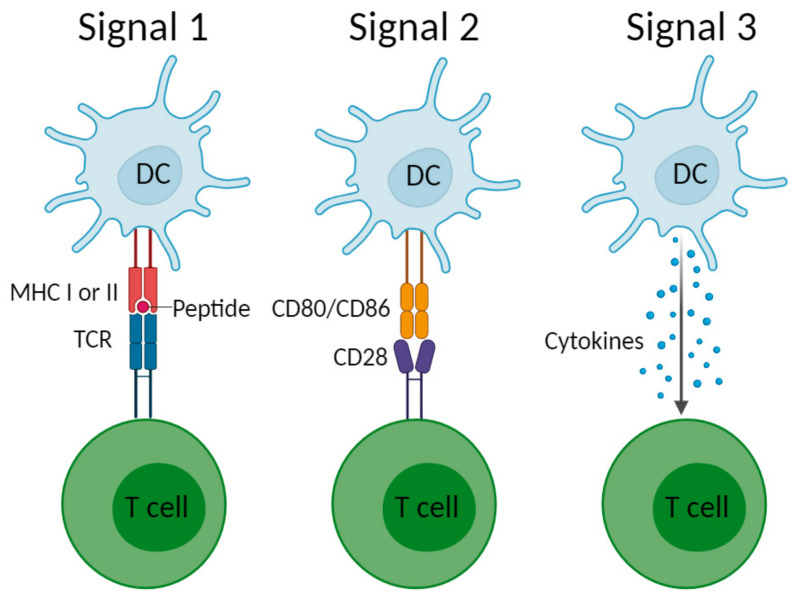
DC-T cell three-signal activation dogma. Abbreviations: CD, cluster of differentiation; DC, dendritic cell; MHC, major histocompatibility complex; TCR, T cell receptor. Created with BioRender.com.

A biomaterial is defined as a material intended to interface with biological systems to evaluate, treat, augment, or replace tissues, organs, or functions of the body [35]. Physical and chemical barriers, such as the skin and stomach acid, respectively, provide the first line of defence against invading pathogens. A breach in these barriers leads to activation of innate immune cells, such as macrophages, dendritic cells (DCs) and neutrophils through pattern recognition receptors (PRRs). PRRs sense conserved molecular structures on microbial surfaces, referred to as pathogen-associated molecular patterns (PAMPs), endogenous danger signals, known as danger-associated molecular patterns (DAMPs), to initiate inflammation [36], or in the case of biomaterials, biomaterial-associated molecular patterns (BAMPs) [37]. The innate immune system acts non-specifically and rapidly, with the aim of immediately clearing the invading pathogen. In contrast, the adaptive immune system is antigen-specific, ensures further clearance of pathogens, and, importantly, provides immunological memory. Originally described by Steinman and Cohn in 1973 [38], DCs are professional antigen-presenting cells that are critical in bridging the innate and adaptive arms of the immune system. Under steady-state conditions, DCs are in an immature differentiation state, with the ability to capture antigens in peripheral tissues, but lacking the ability to efficiently process and present these antigens to T cells [39,40,41]. DC maturation is initiated by the binding of pattern recognition receptors (PRRs), such as toll-like receptors (TLRs), to their respective ligands. Ligands may be exogenously derived PAMPs, such as lipopolysaccharide (LPS), or endogenous DAMPs that are released as a result of cell injury or cell death, such as heat-shock proteins or DNA, respectively [42]. DC maturation is the functional transformation of DCs from antigen-capturing cells to cells with an exceptional capacity for T cell activation, which involves three key signals (Figure 2) and occurs in the lymph nodes. In signal one, DCs present processed antigens to the T cell receptor via major histocompatibility complex (MHC) molecules. Signal 1 is accompanied by a second co-stimulatory signal that is provided by ligation of cluster of differentiation (CD)80/86 to CD28, promoting T cell survival and expansion. In the third signal, the DC provides soluble or membrane-bound cytokines that polarise the functional development of distinct effector T cell subsets, thereby shaping the immune response [43]. The manner in which DCs respond to biomaterials dictates the type of immune response and will be specifically discussed for chitosan-based biomaterials later in the review.

### 2.2. The Foreign Body Response

**Figure 3:**
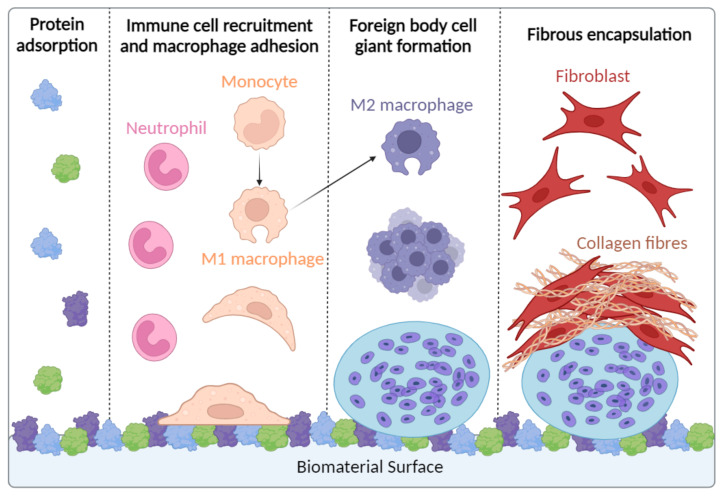
The foreign body response to biomaterials. Biomaterial implantation initiates an acute inflammatory response, collectively referred to as the foreign body response. Figure 3 highlights the key cell types involved and schematically describes the main processes, starting with protein adsorption to the biomaterial and ending with fibrous encapsulation. Created with BioRender.com. Adapted from [44].

Implantation of biomaterials initiates a host inflammatory response, referred to as the foreign body response (FBR) (Figure 3), which has been extensively reviewed [44,45,46,47]. Immediately upon implantation, blood is released from damaged vessels and host serum proteins such as albumin and fibrinogen rapidly adsorb onto the biomaterial surface, leading to aggregation of activated platelets and fibrin. In the case of nanoparticles (NPs), this protein adsorption is referred to as protein corona and affects the physiochemical properties of NPs and their subsequent interaction with biosystems [48]. The resultant provisional matrix is rich in chemotactic and activating molecules, which, in conjunction with DAMPs, cause the recruitment and activation of innate immune cells. The acute inflammatory response is dominated by neutrophils that attempt to destroy the biomaterial through the production of reactive oxygen species (ROS) and proteolytic enzymes, potentiating the immune response and recruiting monocytes. At the site of injury, monocytes differentiate into classically activated or “M1” macrophages, which attempt to degrade the biomaterial before undergoing “frustrated” phagocytosis, ultimately resulting in increased pro-inflammatory cytokine production [44]. Adherent monocytes eventually transition into an alternatively activated or “M2” phenotype, characterised by a reduced degradative capacity, anti-inflammatory cytokine profile, and gained tissue remodelling functionality. Macrophages consequently fuse together to form a foreign body giant cell on the biomaterial surface in an attempt to increase their phagocytic functionality. In the chronic phase of the FBR, excessive deposition of collagen by activated fibroblasts results in the formation of a capsule around the biomaterial in order to prevent further interaction with host tissue [44,45,46,47]. Although the FBR will inevitably be initiated by any biomaterial administration method, it is postulated that the use of minimally invasive injections as opposed to implantation will reduce the extent of DAMPs, and therefore the potency of the response. The Babensee research group investigated whether the PLGA biomaterial form (scaffold vs. microparticle) affected the level of enhancement of the immune response to co-delivered OVA antigen by controlling the total quantities of PLGA and OVA and the release rate of OVA. They found that the level of the humoral immune response was significantly higher and sustained for OVA co-delivered with implanted PLGA scaffolds (0.7 cm diameter, 0.2 cm thick) compared to injected PLGA microparticles (mean diameter 3.5 μm). These results suggest that the FBR associated with biomaterial implantation potentiates the biomaterial adjuvant effect.

### 2.3. Effect of Biomaterial Properties on DC Phenotype

Studies show that the phenotype and resultant function of DCs are dependent on a range of biomaterial physicochemical properties. Shankar et al. investigated how surface chemistries modulate DC phenotype by culturing DCs on self-assembled monolayer (SAM) surfaces of alkanethiols terminated with defined chemical groups, including 1-dodecanethiol (CH_3_ SAM), 11-mercapto-undecanol (OH SAM), 11-mercaptoundecanoic acid (COOH SAM), and 11-amino-1-undecanethiol hydrochloride (NH_2_ SAM). They found that treatment with -OH, -COOH, or -NH_2_ terminated self-assembled monolayer surfaces induced moderate DC maturation, whereas CH_3_ induced least DC maturation [49]. Kou and colleagues also investigated the relationship between the DC phenotype and surface chemistry by co-culturing DCs with a set of 12 polymethacrylates and assessing surface marker expression and cytokine profile. They found that surface carbon is associated with DC maturation, while surface oxygen correlates with an immature DC phenotype [50]. Furthermore, surface hydrophobicity was found to affect DC maturation. Using titanium substrates with different water–air contact angle measurements, Kou et al. reported that surface hydrophobicity induces DC maturation [51]. Similarly, Liu and colleagues treated DCs with morphologically similar synthetic polymeric microparticles that differed in surface hydrophobicity and found that hydrophobicity enhances DC maturation [52]. Another example is in the previously discussed Babensee studies where DCs underwent a greater extent of maturation when cultured on biomaterials that were more hydrophobic, such as PLGA [53,54]. Biomaterial hydrophobicity also increases the adsorption of proteins that support DC adhesion and activation. Surface hydrophilicity is directly related to surface chemistry. For example, increasing the surface oxygen concentration makes a surface more hydrophilic, whereas carbon makes a surface more hydrophobic, which corroborates the finding by Kou et al. [51,55]. Overall, surface hydrophobicity appears to be a key chemical property that causes DC maturation and should therefore be considered in the design of biomaterials.

Although there is limited literature exploring the effect of biomaterial surface charge on the DC phenotype, it is widely accepted that cationic molecules have a greater propensity to interact with negatively charged cell membranes, which in turn may affect their activation and maturation. Using both flow cytometry and confocal microscopy, Foged et al. reported that DC uptake of fluorescently labelled polystyrene microparticles was greatly enhanced when the particles were positively charged [56]. Similarly, Arigita and colleagues showed that only positively charged fluorescently labelled liposomes were taken up by DCs [57]. Ma and colleagues went on to investigate whether increased biomaterial uptake correlated with increased immune responses in DCs. A series of cationic liposomes with different surface densities were prepared, and liposomes with a relatively high charge density potently induced DC maturation in contrast to those with a low charge, which did not affect DC phenotype [58]. These results indicate that although the cationic nature of chitosan promotes cellular uptake, it also induces DC activation. This may adversely affect biomaterial functionality, depending on the application.

Biomaterial surface architecture has also been shown to affect DC immunophenotypes. In a study conducted by van den Dries et al., monocyte-derived dendritic cells (moDCs) were seeded onto 2D and 3D micropatterned silicon surfaces and their phenotypes were compared. Although the expression of maturation and migratory markers was similar between the groups, the expression of MHC class II was significantly higher in DCs cultured on 3D micropatterns, suggesting that dimensionality may play a role in regulating the activation of DCs [59]. The effect of cell culture dimensionality on DC maturation has been investigated in two studies using collagen scaffolds. While one research group reported that DC maturation marker expression and pro-inflammatory cytokine secretion were enhanced in 3D cultures compared to 2D cultures [60], another found that surface marker expression and secreted cytokines of both immature and mature DCs were generally higher in 2D cultures [61]. However, it is important to note that untreated standard tissue culture plates were used as the 2D dimension in both studies; therefore, the observed results may be due to the presence or absence of collagen rather than dimensionality. The influence of biomaterial surface roughness on DC phenotype was also studied by Kou et al. and Zhen et al., who treated DCs with clinical grade titanium with different surface roughness. The substrates induced the same DC phenotype, indicating that surface roughness is not a pivotal factor in regulating DC maturation [51,62]. There appears to be a lack of in-depth studies investigating the role of biomaterial surface architecture, which warrants further investigation. In conclusion, surface hydrophobicity and charge appear to be the major physiochemical properties that modulate the activation and maturation of DCs.

## 3. Endotoxin

**Figure 4:**
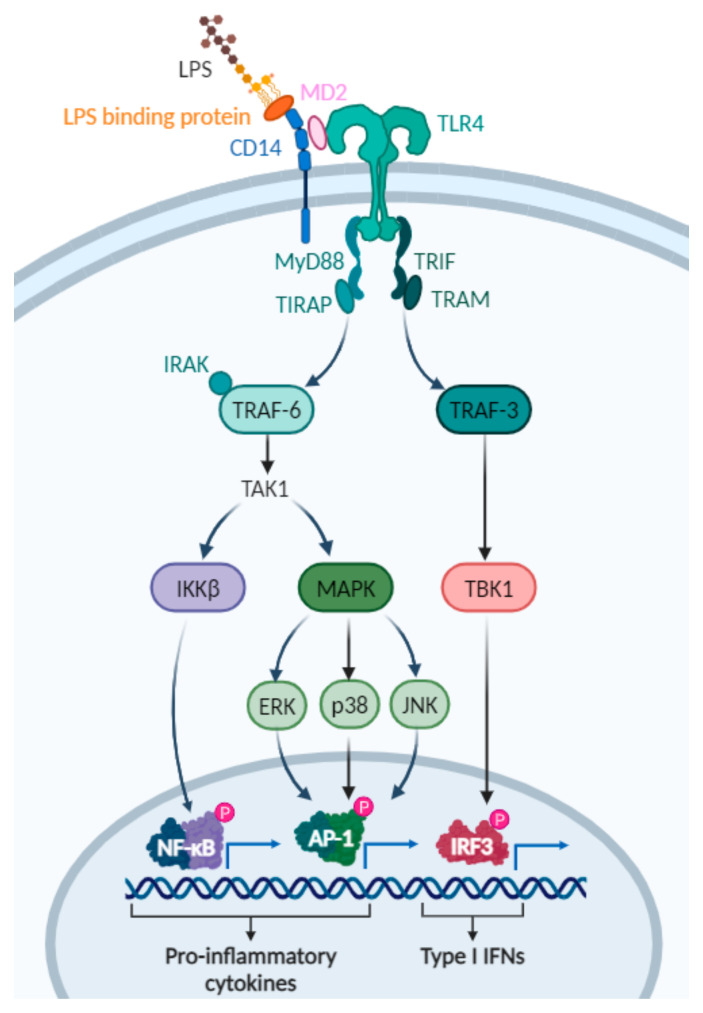
LPS/TLR4 signalling pathways. Upon binding of LPS to the TLR4 complex (TLR4–LBP–CD14–MD2), TLR4 signals through MyD88-dependent and/or MyD88-independent pathways. The MyD88-dependent pathway is initiated by the MyD88 recruitment of IRAKs and TRAF6, which results in activation of TAK1. TAK1 phosphorylates MAPKs (including ERK1/2, p38 and JNK) and the IKKβ kinase complex, leading to transcription of pivotal transcription factors including NF-κB and activator protein-1 (AP-1). These transcription factors translocate to the nucleus and promote the production of inflammatory cytokines. In the MyD88-independent pathway, TRIF recruits TRAF3. TRAF3 activates IRF3 through TBK1, inducing the transcription of type I IFNs and IFN-inducible genes. Abbreviations: AP1, activated protein 1; CD14, cluster of differentiation 14; ERK, extracellular-regulated kinase; IRAK, interleukin receptor-associated kinase; IKK, inhibitor of ĸB kinase; IRF3, interferon response factor 3; JNK, c-Jun N-terminal kinase; LBP, LPS-binding protein; LPS, lipopolysaccharide; MAPK, mitogen-activated protein kinase; MD2, myeloid differentiation factor 2; MyD88, myeloid differentiation primary response protein 88; NF-ĸB, nuclear factor ĸB; p38, protein 38; TAK1, transforming growth factor β-activated kinase 1; TBK1, TANK-binding kinase 1; TIRAP, TIR domain-containing adaptor protein; TRAF, tumour necrosis factor receptor-associated factor; TRAM, TRIF-related adaptor molecule; TRIF, TIR-domain containing adapter-inducing IFN-β; TLR4, toll-like receptor 4. Based on [72]. Created with BioRender.com.

LPSs, also known as endotoxins, are heat-stable molecules that constitute the major structural components of the outer membrane of Gram-negative bacteria [63,64,65]. Bacterial lipopolysaccharide is composed of three parts: lipid A, a core oligosaccharide, and an O-antigen. Lipid A is the hydrophobic, membrane-anchoring region of LPS that consists of glucosamine-based phospholipids. Core oligosaccharides are covalently attached to lipid A through 3-deoxy-D-manno-oct-ulosonic acid and heptose. The O polysaccharide is the hydrophilic, outermost region of LPS. Toxicity is associated with lipid A, while immunogenicity is associated with the polysaccharide components [66]. Immune cells, including DCs, recognise LPS as a PAMP in a TLR4-dependent mechanism. LPS stimulation of TLR4 involves several proteins (Figure 4). First, the LPS-binding protein directly binds LPS and catalyses the transfer of LPS to CD14. CD14 subsequently facilitates the transfer of LPS to the TLR4-myeloid differentiation protein 2 complex [67,68,69,70]. Upon LPS binding, TLR4 undergoes dimerisation and recruits downstream adaptor proteins through interactions with the toll-IL-1 receptor domains. TLR4 signalling is subsequently divided into MyD88-dependent and MyD88-independent (TRIF-dependent) pathways, which mediate pro-inflammatory cytokine expression and induction of type I interferons (IFNs), respectively [71].

### Endotoxin Contamination of Chitosan and Removal Methods

Naturally derived biomaterials, such as alginate and collagen, can potentially be contaminated with endotoxin as they are obtained from non-sterile environments. Synthetic biomaterials can also be contaminated with endotoxins due to contaminated laboratory equipment or reagents [63,65]. Chitosan is particularly susceptible to endotoxin contamination, as its positive charge predisposes its interaction with negatively charged LPS. Despite significant advances in immunomodulatory biomaterial research in recent years, endotoxin contamination is poorly explored within the field [73]. The host immune system can detect and elicit potent immune responses in response to extremely small concentrations of endotoxin, and higher concentrations can induce fever, hypotension, septic shock, respiratory distress syndrome, and other chronic diseases [65]. This has led to the Food and Drug Administration (FDA) enforcing strict regulations on the endotoxin levels in medical devices; the limit is 0.5 EU/mL for products that directly or indirectly contact the cardiovascular system and lymphatic system [74]. DCs are highly sensitive to endotoxins and can be activated by concentrations as low as 0.02 ng/mL (0.1 EU/mL) [75]. As endotoxin is a majorly overlooked problem, many studies investigating the immunomodulatory effect of biomaterials fail to quantify endotoxin contamination, which can skew performance, leading to misleading conclusions [76,77].

Several methods have been developed to remove endotoxins from chitosan; however, endotoxin removal is extremely challenging. Ultrafiltration and size-exclusion chromatography remove endotoxins based on size. Although endotoxins are approximately 10 kDa, they readily form large aggregates of up to 1000 kDa [78,79], making these methods difficult. Most commercially available endotoxin removal resins combine porous cellulose beads for the matrix and cationic poly(ε-lysine), as the affinity ligand. Examples include the Pierce™ High-Capacity Endotoxin Removal Resin and Cellufine™ ETclean. However, endotoxin removal resins are costly and endotoxin removal efficiency is dramatically reduced in viscous and positively charged samples [80], namely chitosan. Ultrasonication and two-phase extraction using detergents are also potential endotoxin removal methods; however, these methods can damage biomaterials, negating their performance [65]. Treatment with acidic and alkaline solutions can also be used to destroy endotoxins [76,81]. While strong alkaline conditions and high temperatures for prolonged periods are used for alkaline chitin deacetylation [82], milder alkaline conditions can be used to remove endotoxins through the hydrolysis of ester and amide linkages found in the lipid A portion of endotoxins [76,81]. As endotoxin is a heat-stable molecule, only extremely high temperatures of 180 °C or above can destroy endotoxin [63,64], which is substantially higher than standard autoclave temperatures (~121 °C). Although high temperatures destroy endotoxin, they induce the Maillard reaction in chitosan, which alters it’s physiochemical structure, meaning it may not be suitable for downstream applications [76,81]. In conclusion, it is imperative that the endotoxin levels of biomaterials are quantified and, if necessary, removed before translation into preclinical and clinical studies.

## 4. Immune Response to Chitin and Chitosan

### 4.1. Interaction with Innate Immune Cells

Chitin, the progenitor of chitosan, is recognised by the mannose receptor, TLRs, and dectin-1 on innate immune cells. The mannose receptor is expressed by human moDCs [83,84,85] and recognises and internalises a range of carbohydrate ligands, including N-acetylglucosamine, one of chitosan’s monomeric units. It is frequently reported in the literature that D-mannose is used to modify chitosan-based particles to promote delivery to immune cells [86,87], confirming this interaction. Although no intracellular signalling motif has been identified at its cytoplasmic tail, the mannose receptor has been shown to be essential for both pro-inflammatory and anti-inflammatory cytokine production [88].

Chitin oligomers have been reported to interact with TLR2 [89,90,91]. TLR2 heterodimers generally initiate a MyD88-dependent intracellular signalling pathway, which modulates gene transcription and ultimately results in pro-inflammatory cytokine production [92]. Interestingly, Fuchs et al. identified chitin chains with six subunits as the smallest immunologically active motif, with those composed of five or fewer subunits being inactive, suggesting that interactions are size-dependent [89,90]. Studies using macrophages from wild-type mice and mice with null mutations of MyD88, TLR2 or TLR4 demonstrated that chitin fragments induce macrophage IL-17 secretion via a MyD88-dependent pathway that involves TLR2, but not TLR4 [90]. Other studies have also demonstrated that chitin-mediated inflammatory responses are abrogated by blocking the chitin–TLR2 interaction [89,93]. Hatase et al. found that the N-acetylglucosamine moiety on the surface of chitin nanofibers was critical for the activation of the TLR2-mediated NF-κB pathway, with immunological response drastically diminished on deacetylated surfaces [91]. TLR4 has also been shown to mediate the stimulating activities of chitosan oligosaccharides. Zhang et al. showed that treatment of RAW264.7 macrophages with anti-murine TLR4 antibody significantly decreases chitosan oligosaccharide-induced pinocytosis and nitric oxide production [94]. To the best of our knowledge, Villiers and colleagues are the only group that have investigated the mechanism by which chitosan interacts with murine DCs and showed that chitosan-induced activation is impaired in DCs derived from TLR4-deficent mice [95]. However, it is important to point out that endotoxin contamination was not reported in this study. Nevertheless, these results suggest that the acetylated unit of chitosan interacts with TLR-2, while the non-acetylated unit reacts with TLR-4.

Dectin-1 is primarily expressed on DCs, and activation promotes phagocytosis, ROS production, and inflammatory cytokine production [96]. Use of the dectin-1 blocker laminarin caused a significant reduction in chitin-induced macrophage TNF-α production [90], showing that this receptor is also important in immune cell recognition of chitosan.

Silva and colleagues showed that different sized chitin fragments interact with different innate immune receptors, which results in the activation of different intracellular signalling pathways in macrophages. Specifically, large chitin fragments (70–100 μm) and extremely small chitin fragments (<2 μm) were found to be inert, whereas intermediate chitin (40–70 μm) and small chitin (<40 μm) stimulated TNF secretion, but only small chitin stimulated the anti-inflammatory cytokine IL-10. They speculated that this size-dependent immunoreactivity resembles the immune response to invading pathogens. Intermediate and small chitin fragments likely induce TNF production to assist in the eradication of the invading pathogen. IL-10 stimulation by small chitin fragments is postulated to be due to down-regulation of the immune response when the pathogen has been eliminated, resulting in cell exposure to smaller chitin fragments [90].

It is important to note that both the degree and pattern of chitosan deacetylation dictate its charge and subsequent cellular interactions. DD directly correlates with increased positivity and hydrophilicity. As cell membranes are negatively charged, it is postulated that the more positively charged a chitosan polymer is, the stronger this interaction will be [97]. Furthermore, heterogeneously deacetylated chitosan further promotes cellular interactions, as there are no interfering N-acetyl groups to interfere with the ionic interactions with the negatively charged cell surface [97]. It is important to highlight that much of the literature focuses on the interactions of immune cells with chitin oligomers. This information is still relevant, as the N-acetylglucosamine units of chitin are part of chitosan’s structure; however, further investigation of how chitosan and chitosan-based hydrogels interact with cells is required. Overall, the literature suggests that chitosan can potentially interact with DCs via multiple receptors and both size and DD are critical parameters in chitosan cellular interactions.

### 4.2. Effect of Chitosan on DC Phenotype

A number of studies have investigated the effect of chitosan on the DC phenotype in vitro (Table 1). The general approach involves culturing immature DCs on chitosan or chitosan hydrogels and measuring cell surface marker expression and cytokine production via flow cytometry and ELISA, respectively. Chitosan has repeatedly been shown to significantly upregulate the expression of human leukocyte antigen (HLA), CD80/86 and CD83 compared to negative controls, indicating that it induces DC maturation [53,54,95,98,99,100]. The results for chitosan-induced cytokine production, migration, and T cell proliferation are, however, variable between studies. Two of five studies reported that the supernatants of DCs cultured on chitosan had significantly higher levels of pro-inflammatory cytokines compared to those of iDCs, with the remaining three studies finding that the cytokine levels were similar between the groups. Oliveira et al. evaluated DC mobility using time-lapse video microscopy and concluded that there was no significant migration of chitosan-treated DCs [98]. Alternatively, Park and Babensee measured DC expression of CD44 migration receptors and found that it was significantly increased compared to iDCs [54]. In order to assess the allostimulatory capacity of chitosan-treated DCs, T cell proliferation was measured in mixed lymphocyte reactions, with the majority of studies finding that they did not induce more T cell proliferation than the control untreated DC. Together, these results suggest that chitosan induces partially mature DCs, lacking the functional ability to stimulate T cells. Table 1 highlights how the chitosan physicochemical properties and endotoxin levels vary between the studies, with several studies omitting this vital information. Furthermore, the forms of chitosan used are different, with some studies using native chitosan and others using crosslinked chitosan hydrogels. All these parameters will undoubtedly affect the interaction and subsequent immunomodulation of DCs. To our knowledge, Ravindranathan and colleagues are the only group who have conducted a comprehensive study investigating the effects of chitosan biochemical properties, namely DD, viscosity, and endotoxin levels, on pro-inflammatory responses by macrophages [101]. First, using chitosan solutions from six different manufacturers, they showed that chitosan from different sources induces differential levels of macrophage TNF-α secretion. Since it was not possible to correlate the effects of any of the properties measured with cytokine production, custom chitosan derivates were obtained from the University of Arkansas Biologics Center (UBAC), which contained undetectable levels of endotoxin (<0.01 EU/mg). UABC chitosan with two different DDs, 80% and 97%, was incubated with macrophages, and no significant difference was found in the TNF-α release. However, it is possible that statistical significance may be observed if a larger range of DD is investigated. Likewise, UABC chitosan with the same DD but three different viscosity ranges (20 to 600 cP) were cultured with macrophages, and no significant difference was observed in the TNF-α production. However, UABC chitosan spiked with varying levels of endotoxin (0.5 and 1 EU) elicited significantly higher levels of TNF-α compared to the untreated control, where there was a positive correlation between endotoxin concentration and TNF-α concentration. Overall, this study shows that neither the DD nor the viscosity of chitosan solution influences macrophage activation, and the level of endotoxin contamination directly affects its TNF-inducing activity. More in-depth studies are required to unravel the effect of chitosan physiochemical properties on immunomodulation of specific cell types, including additional parameters such as pattern of deacetylation, molecular weight, and form (native solution or chitosan-based hydrogels).

### 4.3. Inflammatory Effects of Chitosan

**Figure 5:**
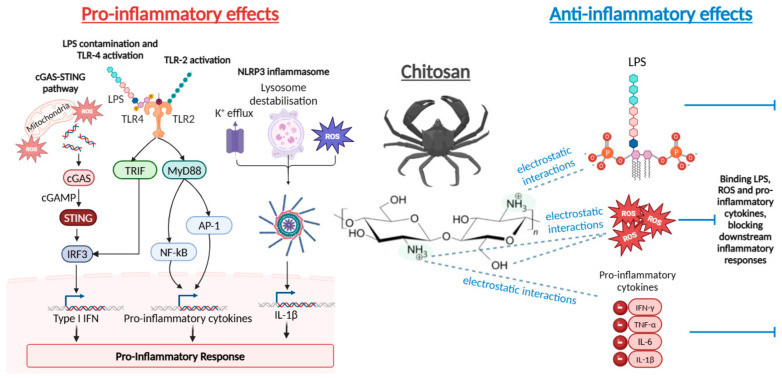
The pro-inflammatory and anti-inflammatory effects of chitosan. Chitosan has been shown to activate mitochondrial disruption, allowing the release of mitochondrial DNA and the production of ROS, leading to activation of the c-GAS-STING pathway and ultimately production of type I IFNs. Chitosan has also been shown to promote the NLPP3 inflammasome through several mechanisms, including potassium ion (K+) efflux, lysosomal destabilisation, and production of ROS, resulting in activation of IL-1β. Also, cationic chitosan is prone to contamination by anionic endotoxins, which can lead to activation of the LPS/TLR-4, also contributing to pro-inflammatory responses. Alternatively, chitosan can also have anti-inflammatory effects in some cases by binding to negatively charged LPS and reducing LPS-induced inflammation, as well as binding negatively charged pro-inflammatory cytokines and reducing downstream pro-inflammatory signalling. Abbreviations: AP-1, activator protein-1; cGAMP, cyclic AMP-GMP; cGAS, cyclic GMP-AMP synthase; IFN, interferon; IL, interleukin; IRF3, interferon response factor 3; LPS, lipopolysaccharide; MyD88, myeloid differentiation primary response protein 88; NF-ĸB, nuclear factor ĸB; ROS, reactive oxygen species; STING, stimulator of interferon genes; TLR, toll-like receptor; TNF, tumour necrosis factor; TRIF, TIR-domain containing adapter-inducing IFN-β. Created with BioRender.com. Chitosan structure extracted from [16].

Chitosan is widely used as an immunostimulatory adjuvant in vaccine research [26,30]; however, its mechanism of action has only recently begun to be elucidated. Caroll et al. identified a novel mechanism of chitosan that involves the activation of the c-GAS-STING pathway in DCs, resulting in the induction of type I IFNs (Figure 5). The pathway involves phagocytic uptake of the polymer, which induces mitochondrial stress, leading to ROS production and the release of mitochondrial DNA. Cyclic GMP-AMP synthase (c-GAS) detects host cytosolic double-stranded DNA, leading to the production of cyclic dinucleotides, which subsequently bind and activate the stimulator of interferon genes (STING). STING directs the activation of the interferon regulatory factor 3 (IRF3) transcription factor, ultimately resulting in type I interferon (IFN) production. The group demonstrated the importance of c-GAS and STING in chitosan-mediated type I IFN production, as cytokine production was completely abrogated in chitosan-stimulated DCs from c-GAS−/− or STING−/− mice [100,102]. Although the endotoxin content of chitosan used in the study was appreciably low (≤100 EU/g), no detailed information was provided regarding the preparation of chitosan. Use of endotoxin-free water and sterilised apparatus is critical in the production of chitosan, as it could otherwise lead to contamination. As LPS has been shown to induce the cGAS-STING pathway [103,104], it cannot be excluded that these results are due to endotoxin contamination of chitosan.

Chitosan has also been shown to activate the NLRP3 inflammasome, which mediates caspase-1 activation and the secretion of pro-inflammatory cytokines IL-1β/IL-18 [105]. However, this study does not report the endotoxin content of the dissolved chitosan used for experimentation. As LPS has been reported to boost activation of the NLRP3 inflammasome [106], endotoxin contamination could indeed be contributing to the observed results. Beuter et al. proposed three mechanisms of chitosan-mediated NLRP3 inflammasome activation, including K^+^ efflux, ROS generation, and lysosomal destabilisation. First, they demonstrated a role for cellular K^+^ efflux by using the K^+^ ion channel inhibitor, glibenclamide, which inhibited IL-1β release in response to chitosan. To analyse the role of ROS, they used the mitochondrial ROS inhibitor Mito-TEMPO and showed that there was a significant reduction in IL-1β release induced by chitosan. Upon phagolysosomal fusion, acidification occurs, which has been shown to be necessary for NLRP3 inflammasome activation. Lysosomal acidification was inhibited with bafilomycin A1, which also caused inhibition of IL-1β [107]. Interestingly, the group also found that while chitosan potently activates the NLRP3 inflammasome, chitin is relatively inert [107], highlighting that chitosan DD may be an important factor in chitosan immunoreactivity. The size of chitosan particles also played an important role, with small particles eliciting the greatest activity. Importantly, the research group removed endotoxins from chitosan and performed experiments under conditions designed to minimise endotoxin contamination, ensuring the observed results were not due to endotoxin contamination. Production of ROS must be a key mechanistic feature of chitosan, given that it is implicated in both the c-GAS-STING pathway and NLRP3 inflammasome activation. Using low-endotoxin chitosan (<500 EU/g), Fong et al. showed that these pathways are mutually exclusive and dose-dependent, with low doses inducing a potent type 1 IFN response in macrophages without activation of the inflammasome and vice versa at higher doses [108].

Turley and colleagues conducted a comprehensive study investigating the effect of chitosan deacetylation and molecular weight on both the cGAS-STING and NLRP3 inflammasome pathways. They generated a panel of chitosan polymers with a range of DDs, from 38% to 100% deacetylation. Only highly deacetylated chitosan polymers were able to drive robust mitochondrial ROS production. To investigate the effect of acetyl group distribution on this pathway, two chitosan polymers were generated with identical DDs but alternative distribution of acetyl groups in which they were either clustered together (heterogeneous) or evenly distributed (homogeneous). While homogenous chitosan polymers failed to drive mitochondrial stress, heterogeneous chitosan significantly enhanced ROS production. Similarly, the group found that highly deacetylated chitosan can promote activation of the NLRP3 inflammasome in vitro in LPS- or CpG-primed DCs, measured by enhanced secretion of IL-1β. Also, heterogenous polymers enhanced IL-1β release, whereas homogenous polymers did not. Importantly, differences in molecular weight had no effect on both mitochondrial stress and IL-1β release [109]. The observed results can partly be explained by the discussion in Section 4.1, which explained that chitosan is more likely to interact with cell membranes if they are more positively charged (increased DD) and have a heterogeneous distribution of acetyl groups. As cellular interactions precede downstream signalling pathways, it follows that chitosan moieties with enhanced binding efficacy result in increased activation of these inflammatory pathways. Therefore, balance needs to be achieved in DD to obtain chitosan with suitable solubility, without compromising degradation or inducing immunoreactivity.

### 4.4. Anti-Inflammatory Effects of Chitosan

Although chitosan-induced inflammatory c-GAS-STING and inflammasome pathways both involve ROS production, chitosan has contradictorily been reported to have antioxidant properties through scavenging free radicals and inhibiting oxidative damage [110]. Rajoka et al. proposed a theory that unstable free radicals may react with the hydroxyl group and amino groups at the C-2, C-3, and C-6 positions of the pyranose ring to produce a stable macromolecule [32]. While antioxidants, including glutathione and catalase levels, were depleted in LPS-induced sepsis, chitosan oligosaccharides were shown to stabilise the redox imbalance [111]. Yoon et al. also found that chitosan oligosaccharides had an antioxidant effect in glycerol-induced acute renal failure through the suppression of glycerol-induced nitric oxide production in the proximal tubules and kidney tissue [112]. Hromis et al. observed that the antioxidant activity of chitosan with 98% DD was 78.5%, while that of chitosan with 90% DD was only 51.5% [113]. While a decrease in deacetylation promotes cellular interactions and thus inflammatory pathways, it also enhances the proportion of amino groups and ROS scavenging capacity. This suggests that the cellular environment dictates whether chitosan adopts pro- or antioxidant activity.

Chitosan is also well renowned for its anti-inflammatory effects via suppression of NF-κB activation. In vitro, chitosan inhibits LPS-induced inflammation through downregulation NF-κB and reduction in the release of pro-inflammatory cytokines in various cell types, including Caco-2 cells [114,115], endothelial cells [116], and RAW 264.7 macrophages [117]. For example, Yoon et al. investigated the effect of chitosan on LPS-stimulated RAW 264.7 cells and found that there was a dose-dependent attenuation of LPS-induced production of TNF-α and IL-6 at both the protein and transcriptional levels [118]. Chitosan has also been shown to have an anti-inflammatory effect in several mouse models of inflammatory diseases, including inflammatory bowel disease [117], ulcerative colitis [119], and autoimmune uveoretinitis [120]. Qiao and colleagues reported that chitosan oligosaccharides are protective against LPS-induced sepsis in mice. Pro-inflammatory markers, including neutrophil infiltration in organs and TNF-α and IL-1β in serum, were significantly reduced by chitosan oligosaccharide treatment [111]. The same research group unravelled the direct target and molecular mechanism of the anti-inflammatory effect of chitosan oligosaccharide on LPS-stimulated cells. They showed that chitosan oligosaccharides significantly inhibit binding of LPS to the TLR4/MD-2 receptor complex, attenuating LPS-induced signal transduction and ultimately decreasing nuclear translocation of NF-κB [121]. Interestingly, Donaldson and colleagues showed that photocrosslinkable gelatin hydrogels “mop up” TNF-α using a cell-free system. In these experiments, recombinant human TNF-α was added to the tissue culture media and incubated with hydrogels for 4 h, and supernatants were collected for cytokine analysis. There was a significant reduction in TNF-α detected in hydrogel-coated wells compared to untreated wells [122]. In an LPS-free study, Mohyuddin et al. demonstrated that chitosan has a protective effect on immune function in heat-stressed mice. Heat stress led to an increase in serum pro-inflammatory cytokines (IL-10, IL-6, and TNF-α) in mice; however, oral administration of chitosan significantly decreased pro-inflammatory cytokine response compared with the heat stress control group on days 1, 7, and 14 of the experiment. The results showed that chitosan has an excellent anti-inflammatory capability, which inhibits the level of pro-inflammatory cytokines [123]. According to Messina and colleagues, the majority of pro-inflammatory cytokines are negatively charged. It can therefore be concluded that chitosan’s anti-inflammatory effects are likely due to its interaction with negatively charged LPS or cell-secreted pro-inflammatory cytokines (Figure 5).

Paradoxically, low molecular weight chitosan is reported to have immunostimulatory activity. The Qu research group showed that low molecular weight chitosan (3 kDa and 50 kDa) elicited potent immunostimulatory responses in RAW264.7 macrophages co-treated with LPS and chitosan by promoting the expression of genes and key molecules in the NF-κB and AP-1 pathways, including IKKβ, TRAF6, and JNK1, in which there was a correlation between decrease in molecular weight and potency of responses [124,125]. By investigating a larger range of molecular weight chitosans, Chang et al. found that chitosan has dual activity in the LPS-induced murine RAW 264.7 macrophage phenotype. Larger molecular weight chitosan derivatives (156, 72 kDa) downregulated NF-κB activation, leading to anti-inflammatory effects, including abrogating pro-inflammatory cytokine production and decreasing nitric oxide production. On the other hand, low molecular weight chitosan (7.1 kDa) upregulated components of the NF-κB activation and subsequently enhanced the production of TNF-α, IL-6, and nitric oxide. Similarly, Niu et al. found that chitosan had anti-inflammatory activity and modulation of intestinal microflora in an ulcerative colitis model, where higher molecular weight chitosan had a better therapeutic effect [126]. However, it is important to note that in this study, cells were pre-treated with dexamethasone, so the observed anti-inflammatory effects may not be due to chitosan exclusively. Interestingly, the molecular weight of chitosan affects its complexation with LPS, resulting in a differential reduction in toxicity. A counterintuitive observation was made by Davyoda et al., who showed that chitosan with low molecular weight (20 kD) has a higher affinity to LPS compared to chitosan with high molecular weight (140 kD). While low molecular weight chitosan has flexible chains, high molecular weight chitosan has a rigid asymmetrical structure. As a result, high molecular weight chitosan localises on the surface of endotoxin aggregates and low molecular weight chitosan penetrates into the aggregates [127]. The group also studied the effect of chitosan molecular weight on the ability of LPS to interact with the neutrophils of human whole blood. Although chitosan-LPS complexes composed of high molecular weight chitosan (110 kDa) reacted less with the cells, they were more effective at inhibiting the pro-inflammatory cytokine-inducing activity of LPS compared to low molecular weight chitosan (5 kDa). Furthermore, consistent with previous studies, low molecular weight chitosan induced a twofold increase in TNF-α production relative to high molecular weight chitosan [128]. By studying the supramolecular structures of LPS-chitosan complexes formed with LPS from different bacterial sources (*Escherichia coli* O55:B5 and Yersinia pseudotuberculosis 1B 598) using atomic force microscopy [129], the group showed that the macromolecular organisation of endotoxins also affects chitosan–endotoxin interactions. Together, these data suggest that the physiochemical properties of chitosan and the presence of LPS—whether that be through LPS contamination of chitosan, LPS present in an inflammatory cellular environment, or LPS added as a reagent in in vitro studies—are key interplaying factors that affect chitosan’s immunomodulatory activity. The properties chitosan should therefore be carefully considered and recorded in scientific publications. Furthermore, endotoxin quantification and removal should be a prerequisite when developing chitosan-based hydrogels for biomedical applications.

**Table 1:** The effect of chitosan on DC phenotype.

DC	Chitosan	Negative Control	Positive Control	HLA	CD80/ CD86	CD83	Cytokines	Migration	T Cell Proliferation	Endotoxin (EU/mL)	Reference
Human moDC	400 kDa	Untreated	LPS-treated	↑ HLA-DQ	↑	NA	NA	NA	NA	1.58 (chitosan disk)	[53]
Human moDC	324 kDa DD 89%	Untreated	LPS-treated	↑ HLA-DRQ	↑	—	↑ TNF-α ↑ IL-1β — IL-6 — IL-12 — IL-23 — TGF-β ↓ IL-10	—	—	N/A	[99]
Human moDC	400 kDa DD ≥ 75%	Untreated	LPS-treated	↑ HLA-DR	↑	↑	↑ TNF-α ↑ IL-6	↑	↑	0.0007 (chitosan film)	[54]
Murine BM-DC	612 kDa	Untreated	LPS-treated	↑ MHC II	↑	NA	— TNF — IL-12 — IL-10 — IL-1β — IL-6	NA	—	N/A	[95]
Human moDC	DD > 85%	Untreated	TNFα + IL-1β, IL-6 + PGE2 treatment	— HLA-DRQ	—	—	NA	NA	NA	<0.6 (lyophilised microparticles)	[100]
Murine BM-DC	>150 kDa DD 75–90%	Untreated	LPS-treated	NA	↑	NA	— IL-6 — IL-12p40	NA	NA	≤0.1 (powder, measured by manufacturer)	[98]

The phenotypes of DCs treated with chitosan are shown by statistical significance compared with immature DCs, where a *p* value ≤ 0.05 is considered to be significant. ↑, significantly higher; —, no significant difference; ↓, significantly lower. Abbreviations: BM, bone marrow-derived; DC, dendritic cell; DD, degree of deacetylation; EU, endotoxin unit; HLA, human leukocyte antigen; IL, interleukin; LPS, lipopolysaccharide; MHC, major histocompatibility complex; kDa, kilodalton; MO, monocyte-derived; NA, not assessed; TGF, transforming growth factor; TNF, tumour necrosis factor. Information from [53,54,95,98,99,100].

## 5. Conclusions

In conclusion, chitosan is an extremely versatile material with inherent biological and chemical properties, including biocompatibility, biodegradability, mucoadhesion, and nontoxicity, making it useful for a wide range of biomedical applications. Depending on the medical application, a pro- or anti-inflammatory response to chitosan may be desired. For example, inflammatory adjuvant activity is preferred in vaccine therapy, whereas induction of an inflammatory environment is undesirable in transplantation, as it may promote rejection. It is extremely concerning how divergent the literature is regarding the nature and potency of immune responses initiated by chitosan. There are many variables in the experimental design that may drastically influence the immunoreactivity of chitosan. Chitosan is produced from chitin using different treatment regimens, leading to batch-to-batch variation in physiochemical properties, including molecular weight, polydispersity, and DD, which drastically affect immune responses. At the same time, material datasheets currently offered by manufacturers do not provide sufficient detail that would clarify variations at the desired level. It is important to note that the cellular models used (e.g., cell type, primary or cell line, murine or human origin) may also influence the immune response to chitosan. As chitosan is a naturally derived polymer, it may contain contaminants, including heavy metals and endotoxins. Endotoxin contamination is a particular concern for chitosan, as the cationic nature of the polymer promotes its interaction with the negatively charged phosphate groups in LPS. Endotoxin contamination is a majorly overlooked issue in the field of biomaterials, with many researchers omitting endotoxin quantification from their studies. Furthermore, there is no standardised method to measure endotoxins in chitosan, with some studies using solution and others using the media incubated with hydrogels, leading to discrepancies in results. Overall, both differences in physiochemical properties and endotoxin contamination have ultimately led to divergent opinions as to whether chitosan is a pro-inflammatory or anti-inflammatory polymer. It is likely that the immune response induced by chitosan is also dictated by environmental conditions. When chitosan is administered to an inflammatory environment, it is possible that chitosan preferentially binds to LPS or pro-inflammatory cytokines due to strong electrostatic attraction, ultimately preventing downstream pro-inflammatory pathways. On the other hand, when immune cells are treated with chitosan in homeostatic conditions, chitosan can interact with innate immune cells through various PRRs, resulting in their activation. Overall, to advance research efforts, facilitate the clinical translation of chitosan, and avoid confusion arising from data interpretation, it is imperative that chitosan sources provide a comprehensive certificate of analysis and that research articles disclose the properties and endotoxin content of the chitosan used.
